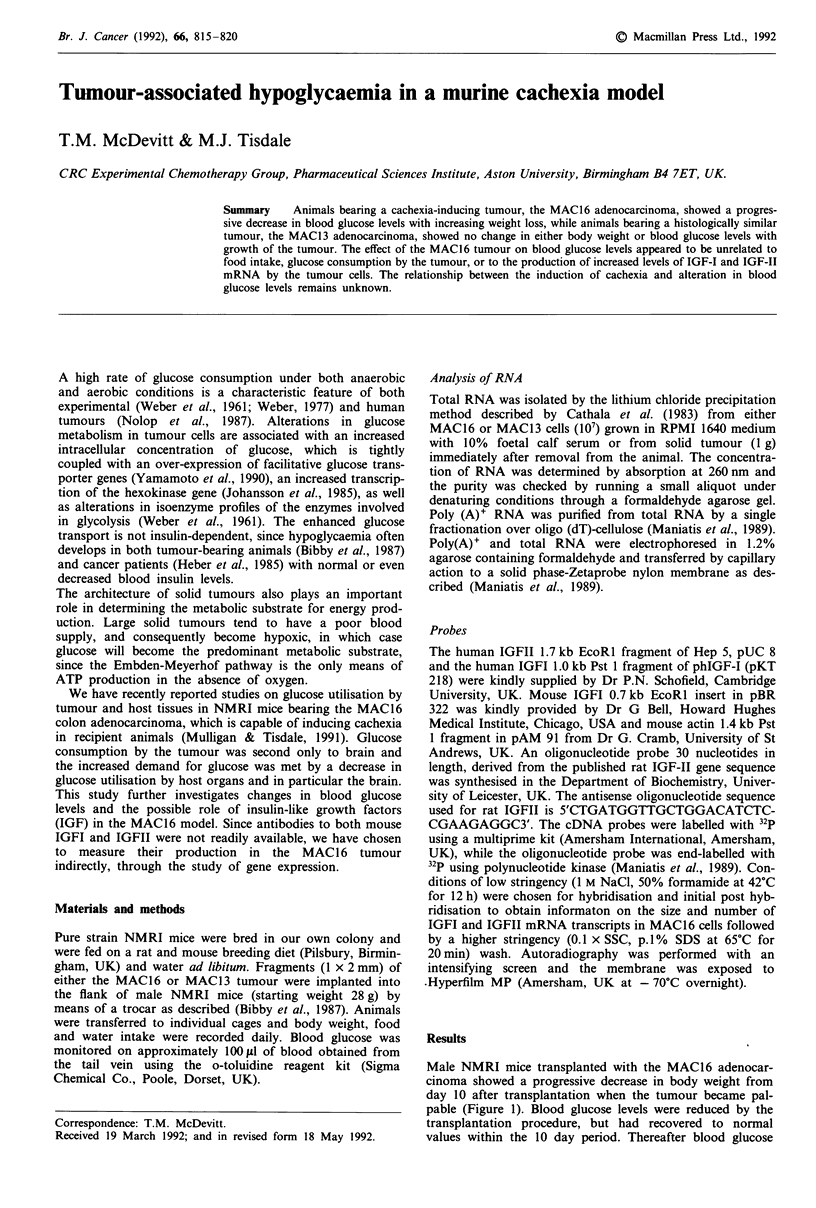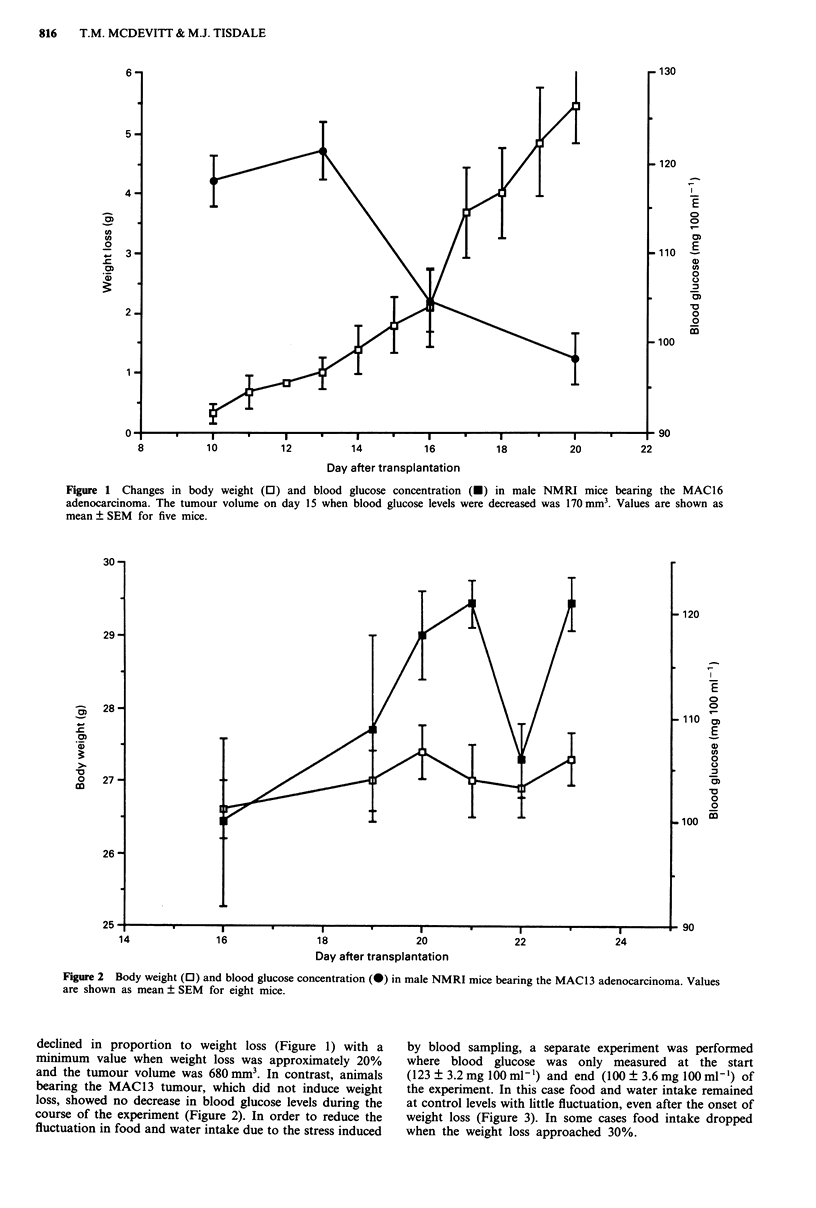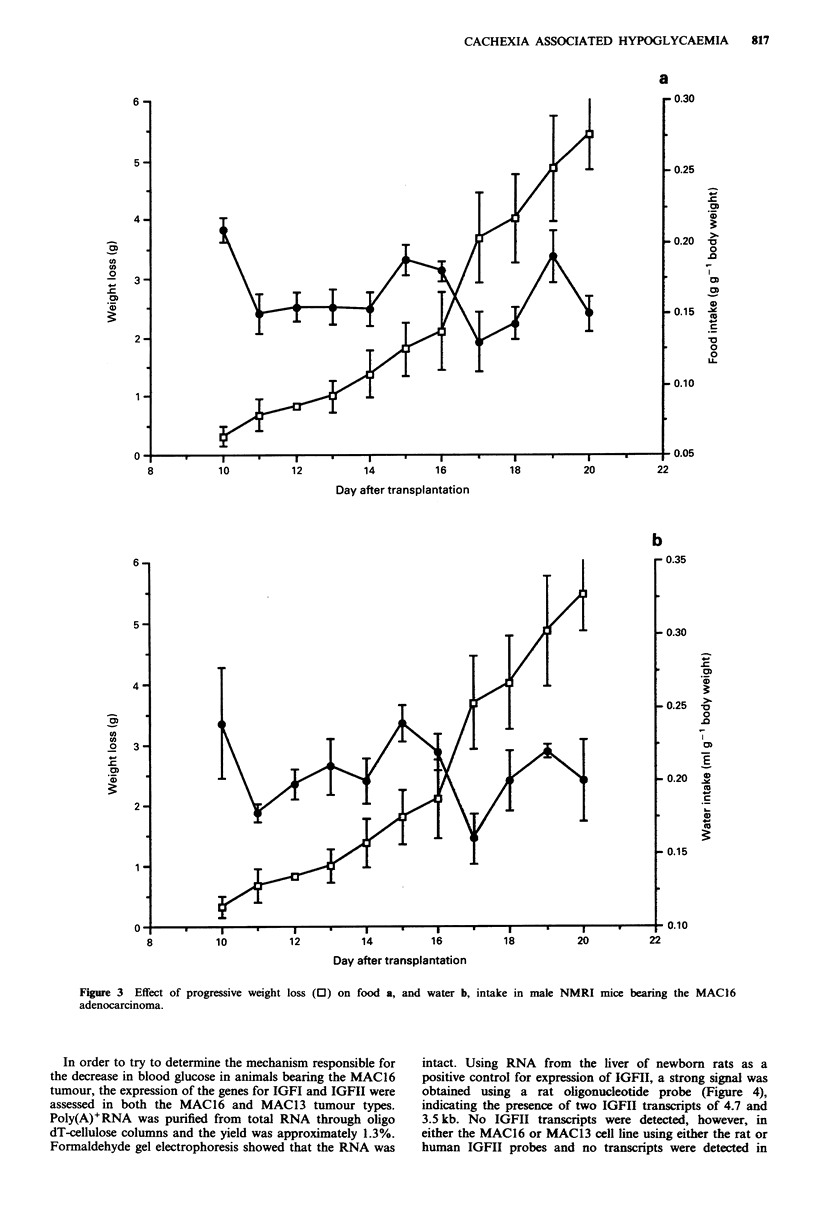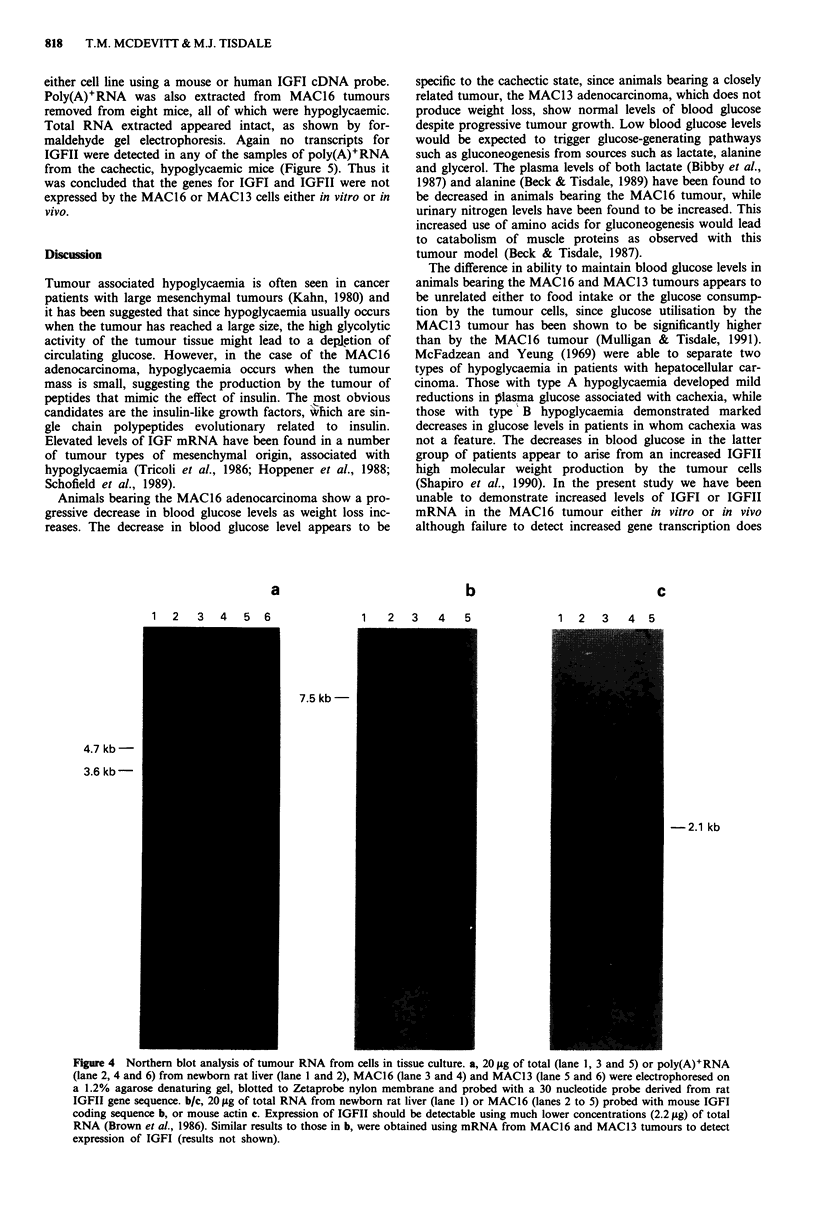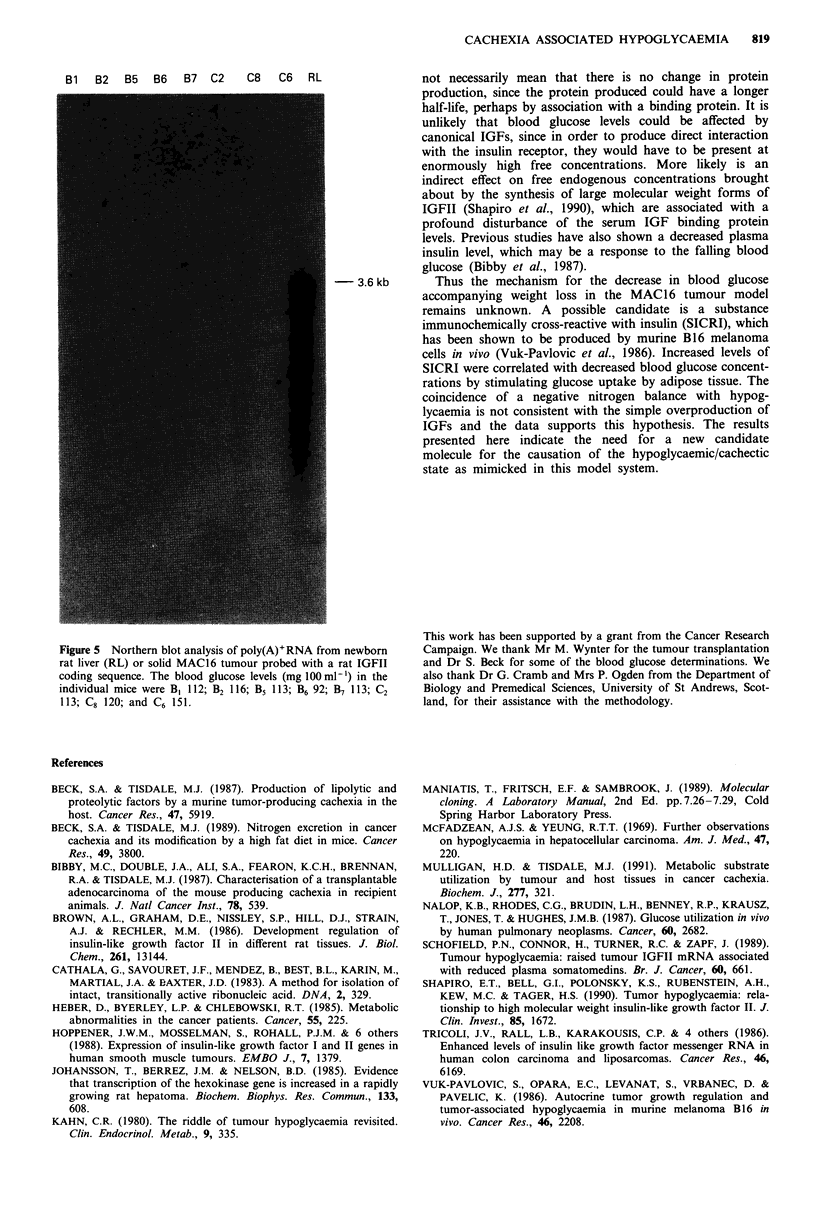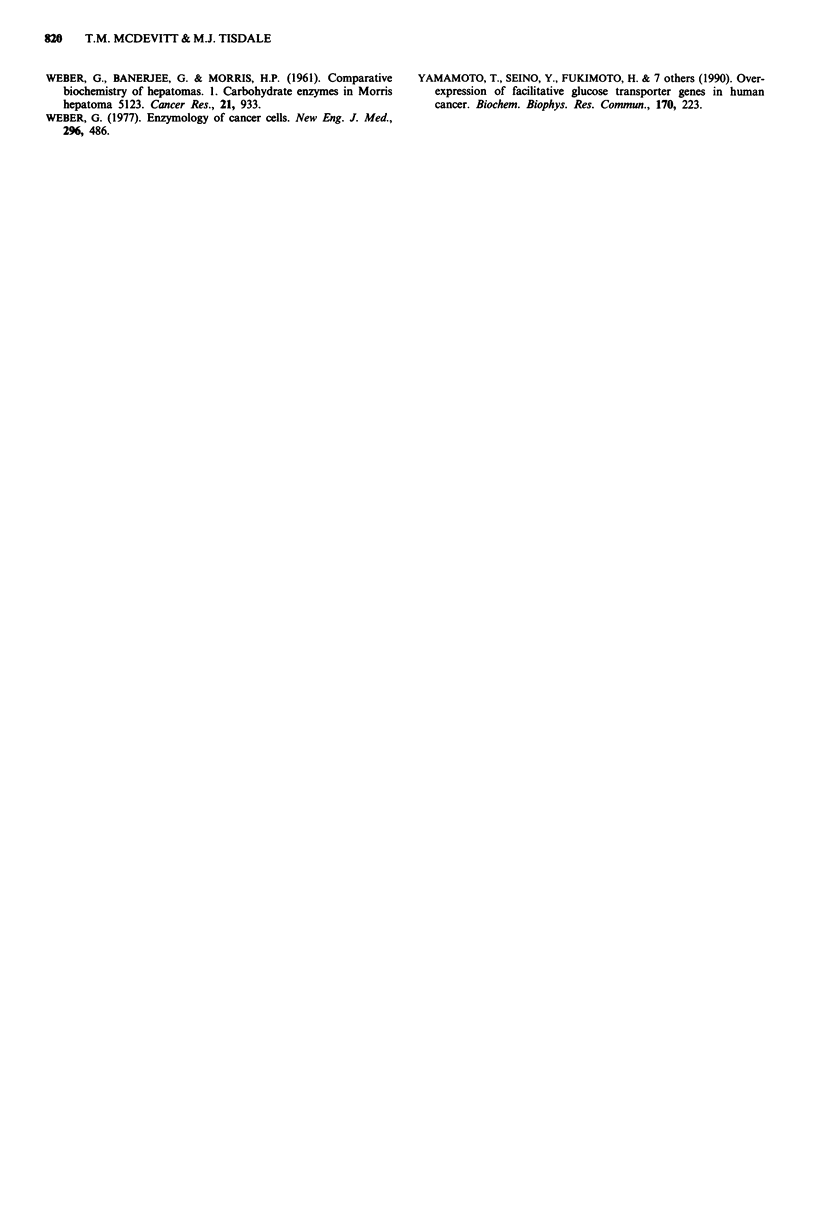# Tumour-associated hypoglycaemia in a murine cachexia model.

**DOI:** 10.1038/bjc.1992.366

**Published:** 1992-11

**Authors:** T. M. McDevitt, M. J. Tisdale

**Affiliations:** CRC Experimental Chemotherapy Group, Pharmaceutical Sciences Institute, Aston University, Birmingham, UK.

## Abstract

**Images:**


Br. J. Cancer (1992), 66, 815–820 © Macmillan Press Ltd., 1992

### Tumour-associated hypoglycaemia in a murine cachexia model

T.M. McDevitt & M.J. Tisdale

*CRC Experimental Chemotherapy Group, Pharmaceutical Sciences Institute, Aston University, Birmingham B4 7ET, UK.*

**Summary** Animals bearing a cachexia-inducing tumour, the MAC16 adenocarcinoma, showed a progressive decrease in blood glucose levels with increasing weight loss, while animals bearing a histologically similar tumour, the MAC13 adenocarcinoma, showed no change in either body weight or blood glucose levels with growth of the tumour. The effect of the MAC16 tumour on blood glucose levels appeared to be unrelated to food intake, glucose consumption by the tumour, or to the production of increased levels of IGF-I and IGF-II mRNA by the tumour cells. The relationship between the induction of cachexia and alteration in blood glucose levels remains unknown.

A high rate of glucose consumption under both anaerobic and aerobic conditions is a characteristic feature of both experimental (Weber *et al.*, 1961; Weber, 1977) and human tumours (Nolop *et al.*, 1987). Alterations in glucose metabolism in tumour cells are associated with an increased intracellular concentration of glucose, which is tightly coupled with an over-expression of facilitative glucose transporter genes (Yamamoto *et al.*, 1990), an increased transcription of the hexokinase gene (Johansson *et al.*, 1985), as well as alterations in isoenzyme profiles of the enzymes involved in glycolysis (Weber *et al.*, 1961). The enhanced glucose transport is not insulin-dependent, since hypoglycaemia often develops in both tumour-bearing animals (Bibby *et al.*, 1987) and cancer patients (Heber *et al.*, 1985) with normal or even decreased blood insulin levels.

The architecture of solid tumours also plays an important role in determining the metabolic substrate for energy production. Large solid tumours tend to have a poor blood supply, and consequently become hypoxic, in which case glucose will become the predominant metabolic substrate, since the Embden-Meyerhof pathway is the only means of ATP production in the absence of oxygen.

We have recently reported studies on glucose utilisation by tumour and host tissues in NMRI mice bearing the MAC16 colon adenocarcinoma, which is capable of inducing cachexia in recipient animals (Mulligan & Tisdale, 1991). Glucose consumption by the tumour was second only to brain and the increased demand for glucose was met by a decrease in glucose utilisation by host organs and in particular the brain. This study further investigates changes in blood glucose levels and the possible role of insulin-like growth factors (IGF) in the MAC16 model. Since antibodies to both mouse IGFI and IGFII were not readily available, we have chosen to measure their production in the MAC16 tumour indirectly, through the study of gene expression.

### Materials and methods

Pure strain NMRI mice were bred in our own colony and were fed on a rat and mouse breeding diet (Pilsbury, Birmingham, UK) and water *ad libitum.* Fragments (1 × 2 mm) of either the MAC16 or MAC13 tumour were implanted into the flank of male NMRI mice (starting weight 28 g) by means of a trocar as described (Bibby *et al.*, 1987). Animals were transferred to individual cages and body weight, food and water intake were recorded daily. Blood glucose was monitored on approximately 100 μl of blood obtained from the tail vein using the o-toluidine reagent kit (Sigma Chemical Co., Poole, Dorset, UK).

Correspondence: T.M. McDevitt.
Received 19 March 1992; and in revised form 18 May 1992.

### *Analysis of RNA*

Total RNA was isolated by the lithium chloride precipitation method described by Cathala *et al.* (1983) from either MAC16 or MAC13 cells ($10^7$) grown in RPMI 1640 medium with 10% foetal calf serum or from solid tumour (1 g) immediately after removal from the animal. The concentration of RNA was determined by absorption at 260 nm and the purity was checked by running a small aliquot under denaturing conditions through a formaldehyde agarose gel. Poly $(A)^+$ RNA was purified from total RNA by a single fractionation over oligo (dT)-cellulose (Maniatis *et al.*, 1989). Poly$(A)^+$ and total RNA were electrophoresed in 1.2% agarose containing formaldehyde and transferred by capillary action to a solid phase-Zetaprobe nylon membrane as described (Maniatis *et al.*, 1989).

### *Probes*

The human IGFII 1.7 kb EcoR1 fragment of Hep 5, pUC 8 and the human IGFI 1.0 kb Pst 1 fragment of phIGF-I (pKT 218) were kindly supplied by Dr P.N. Schofield, Cambridge University, UK. Mouse IGFI 0.7 kb EcoR1 insert in pBR 322 was kindly provided by Dr G Bell, Howard Hughes Medical Institute, Chicago, USA and mouse actin 1.4 kb Pst 1 fragment in pAM 91 from Dr G. Cramb, University of St Andrews, UK. An oligonucleotide probe 30 nucleotides in length, derived from the published rat IGF-II gene sequence was synthesised in the Department of Biochemistry, University of Leicester, UK. The antisense oligonucleotide sequence used for rat IGFII is 5'CTGATGGTTGCTGGACATCTCCGAAGAGGC3'. The cDNA probes were labelled with $^{32}P$ using a multiprime kit (Amersham International, Amersham, UK), while the oligonucleotide probe was end-labelled with $^{32}P$ using polynucleotide kinase (Maniatis *et al.*, 1989). Conditions of low stringency (1 M NaCl, 50% formamide at 42°C for 12 h) were chosen for hybridisation and initial post hybridisation to obtain informaton on the size and number of IGFI and IGFII mRNA transcripts in MAC16 cells followed by a higher stringency (0.1 × SSC, p.1% SDS at 65°C for 20 min) wash. Autoradiography was performed with an intensifying screen and the membrane was exposed to Hyperfilm MP (Amersham, UK at − 70°C overnight).

### Results

Male NMRI mice transplanted with the MAC16 adenocarcinoma showed a progressive decrease in body weight from day 10 after transplantation when the tumour became palpable (Figure 1). Blood glucose levels were reduced by the transplantation procedure, but had recovered to normal values within the 10 day period. Thereafter blood glucose

Figure 1 Changes in body weight (□) and blood glucose concentration (■) in male NMRI mice bearing the MAC16 adenocarcinoma. The tumour volume on day 15 when blood glucose levels were decreased was 170 $mm^3$. Values are shown as mean ± SEM for five mice.

Figure 2 Body weight (□) and blood glucose concentration (●) in male NMRI mice bearing the MAC13 adenocarcinoma. Values are shown as mean ± SEM for eight mice.

declined in proportion to weight loss (Figure 1) with a minimum value when weight loss was approximately 20% and the tumour volume was 680 $mm^3$. In contrast, animals bearing the MAC13 tumour, which did not induce weight loss, showed no decrease in blood glucose levels during the course of the experiment (Figure 2). In order to reduce the fluctuation in food and water intake due to the stress induced by blood sampling, a separate experiment was performed where blood glucose was only measured at the start (123 ± 3.2 mg 100 $ml^{-1}$) and end (100 ± 3.6 mg 100 $ml^{-1}$) of the experiment. In this case food and water intake remained at control levels with little fluctuation, even after the onset of weight loss (Figure 3). In some cases food intake dropped when the weight loss approached 30%.

**Figure 3** Effect of progressive weight loss (□) on food **a**, and water **b**, intake in male NMRI mice bearing the MAC16 adenocarcinoma.

In order to try to determine the mechanism responsible for the decrease in blood glucose in animals bearing the MAC16 tumour, the expression of the genes for IGFI and IGFII were assessed in both the MAC16 and MAC13 tumour types. Poly(A)$^+$RNA was purified from total RNA through oligo dT-cellulose columns and the yield was approximately 1.3%. Formaldehyde gel electrophoresis showed that the RNA was intact. Using RNA from the liver of newborn rats as a positive control for expression of IGFII, a strong signal was obtained using a rat oligonucleotide probe (Figure 4), indicating the presence of two IGFII transcripts of 4.7 and 3.5 kb. No IGFII transcripts were detected, however, in either the MAC16 or MAC13 cell line using either the rat or human IGFII probes and no transcripts were detected in either cell line using a mouse or human IGFI cDNA probe. Poly(A)$^+$RNA was also extracted from MAC16 tumours removed from eight mice, all of which were hypoglycaemic. Total RNA extracted appeared intact, as shown by formaldehyde gel electrophoresis. Again no transcripts for IGFII were detected in any of the samples of poly(A)$^+$RNA from the cachectic, hypoglycaemic mice (Figure 5). Thus it was concluded that the genes for IGFI and IGFII were not expressed by the MAC16 or MAC13 cells either *in vitro* or *in vivo*.

### Discussion

Tumour associated hypoglycaemia is often seen in cancer patients with large mesenchymal tumours (Kahn, 1980) and it has been suggested that since hypoglycaemia usually occurs when the tumour has reached a large size, the high glycolytic activity of the tumour tissue might lead to a depletion of circulating glucose. However, in the case of the MAC16 adenocarcinoma, hypoglycaemia occurs when the tumour mass is small, suggesting the production by the tumour of peptides that mimic the effect of insulin. The most obvious candidates are the insulin-like growth factors, which are single chain polypeptides evolutionary related to insulin. Elevated levels of IGF mRNA have been found in a number of tumour types of mesenchymal origin, associated with hypoglycaemia (Tricoli *et al.*, 1986; Hoppener *et al.*, 1988; Schofield *et al.*, 1989).

Animals bearing the MAC16 adenocarcinoma show a progressive decrease in blood glucose levels as weight loss increases. The decrease in blood glucose level appears to be specific to the cachectic state, since animals bearing a closely related tumour, the MAC13 adenocarcinoma, which does not produce weight loss, show normal levels of blood glucose despite progressive tumour growth. Low blood glucose levels would be expected to trigger glucose-generating pathways such as gluconeogenesis from sources such as lactate, alanine and glycerol. The plasma levels of both lactate (Bibby *et al.*, 1987) and alanine (Beck & Tisdale, 1989) have been found to be decreased in animals bearing the MAC16 tumour, while urinary nitrogen levels have been found to be increased. This increased use of amino acids for gluconeogenesis would lead to catabolism of muscle proteins as observed with this tumour model (Beck & Tisdale, 1987).

The difference in ability to maintain blood glucose levels in animals bearing the MAC16 and MAC13 tumours appears to be unrelated either to food intake or the glucose consumption by the tumour cells, since glucose utilisation by the MAC13 tumour has been shown to be significantly higher than by the MAC16 tumour (Mulligan & Tisdale, 1991). McFadzean and Yeung (1969) were able to separate two types of hypoglycaemia in patients with hepatocellular carcinoma. Those with type A hypoglycaemia developed mild reductions in plasma glucose associated with cachexia, while those with type B hypoglycaemia demonstrated marked decreases in glucose levels in patients in whom cachexia was not a feature. The decreases in blood glucose in the latter group of patients appear to arise from an increased IGFII high molecular weight production by the tumour cells (Shapiro *et al.*, 1990). In the present study we have been unable to demonstrate increased levels of IGFI or IGFII mRNA in the MAC16 tumour either *in vitro* or *in vivo* although failure to detect increased gene transcription does

**Figure 4** Northern blot analysis of tumour RNA from cells in tissue culture. **a**, 20 μg of total (lane 1, 3 and 5) or poly(A)$^+$RNA (lane 2, 4 and 6) from newborn rat liver (lane 1 and 2), MAC16 (lane 3 and 4) and MAC13 (lane 5 and 6) were electrophoresed on a 1.2% agarose denaturing gel, blotted to Zetaprobe nylon membrane and probed with a 30 nucleotide probe derived from rat IGFII gene sequence. **b/c**, 20 μg of total RNA from newborn rat liver (lane 1) or MAC16 (lanes 2 to 5) probed with mouse IGFI coding sequence **b**, or mouse actin **c**. Expression of IGFII should be detectable using much lower concentrations (2.2 μg) of total RNA (Brown *et al.*, 1986). Similar results to those in **b**, were obtained using mRNA from MAC16 and MAC13 tumours to detect expression of IGFI (results not shown).

**Figure 5** Northern blot analysis of poly(A)$^+$RNA from newborn rat liver (RL) or solid MAC16 tumour probed with a rat IGFII coding sequence. The blood glucose levels (mg 100 ml$^{-1}$) in the individual mice were $B_1$ 112; $B_2$ 116; $B_5$ 113; $B_6$ 92; $B_7$ 113; $C_2$ 113; $C_8$ 120; and $C_6$ 151.

not necessarily mean that there is no change in protein production, since the protein produced could have a longer half-life, perhaps by association with a binding protein. It is unlikely that blood glucose levels could be affected by canonical IGFs, since in order to produce direct interaction with the insulin receptor, they would have to be present at enormously high free concentrations. More likely is an indirect effect on free endogenous concentrations brought about by the synthesis of large molecular weight forms of IGFII (Shapiro *et al.*, 1990), which are associated with a profound disturbance of the serum IGF binding protein levels. Previous studies have also shown a decreased plasma insulin level, which may be a response to the falling blood glucose (Bibby *et al.*, 1987).

Thus the mechanism for the decrease in blood glucose accompanying weight loss in the MAC16 tumour model remains unknown. A possible candidate is a substance immunochemically cross-reactive with insulin (SICRI), which has been shown to be produced by murine B16 melanoma cells *in vivo* (Vuk-Pavlovic *et al.*, 1986). Increased levels of SICRI were correlated with decreased blood glucose concentrations by stimulating glucose uptake by adipose tissue. The coincidence of a negative nitrogen balance with hypoglycaemia is not consistent with the simple overproduction of IGFs and the data supports this hypothesis. The results presented here indicate the need for a new candidate molecule for the causation of the hypoglycaemic/cachectic state as mimicked in this model system.

This work has been supported by a grant from the Cancer Research Campaign. We thank Mr M. Wynter for the tumour transplantation and Dr S. Beck for some of the blood glucose determinations. We also thank Dr G. Cramb and Mrs P. Ogden from the Department of Biology and Premedical Sciences, University of St Andrews, Scotland, for their assistance with the methodology.

### References

BECK, S.A. & TISDALE, M.J. (1987). Production of lipolytic and proteolytic factors by a murine tumor-producing cachexia in the host. *Cancer Res.*, **47**, 5919.

BECK, S.A. & TISDALE, M.J. (1989). Nitrogen excretion in cancer cachexia and its modification by a high fat diet in mice. *Cancer Res.*, **49**, 3800.

BIBBY, M.C., DOUBLE, J.A., ALI, S.A., FEARON, K.C.H., BRENNAN, R.A. & TISDALE, M.J. (1987). Characterisation of a transplantable adenocarcinoma of the mouse producing cachexia in recipient animals. *J. Natl Cancer Inst.*, **78**, 539.

BROWN, A.L., GRAHAM, D.E., NISSLEY, S.P., HILL, D.J., STRAIN, A.J. & RECHLER, M.M. (1986). Development regulation of insulin-like growth factor II in different rat tissues. *J. Biol. Chem.*, **261**, 13144.

CATHALA, G., SAVOURET, J.F., MENDEZ, B., BEST, B.L., KARIN, M., MARTIAL, J.A. & BAXTER, J.D. (1983). A method for isolation of intact, transitionally active ribonucleic acid. *DNA*, **2**, 329.

HEBER, D., BYERLEY, L.P. & CHLEBOWSKI, R.T. (1985). Metabolic abnormalities in the cancer patients. *Cancer*, **55**, 225.

HOPPENER, J.W.M., MOSSELMAN, S., ROHALL, P.J.M. & 6 others (1988). Expression of insulin-like growth factor I and II genes in human smooth muscle tumours. *EMBO J.*, **7**, 1379.

JOHANSSON, T., BERREZ, J.M. & NELSON, B.D. (1985). Evidence that transcription of the hexokinase gene is increased in a rapidly growing rat hepatoma. *Biochem. Biophys. Res. Commun.*, **133**, 608.

KAHN, C.R. (1980). The riddle of tumour hypoglycaemia revisited. *Clin. Endocrinol. Metab.*, **9**, 335.

MANIATIS, T., FRITSCH, E.F. & SAMBROOK, J. (1989). *Molecular cloning. A Laboratory Manual*, 2nd Ed. pp.7.26–7.29, Cold Spring Harbor Laboratory Press.

MCFADZEAN, A.J.S. & YEUNG, R.T.T. (1969). Further observations on hypoglycaemia in hepatocellular carcinoma. *Am. J. Med.*, **47**, 220.

MULLIGAN, H.D. & TISDALE, M.J. (1991). Metabolic substrate utilization by tumour and host tissues in cancer cachexia. *Biochem. J.*, **277**, 321.

NALOP, K.B., RHODES, C.G., BRUDIN, L.H., BENNEY, R.P., KRAUSZ, T., JONES, T. & HUGHES, J.M.B. (1987). Glucose utilization *in vivo* by human pulmonary neoplasms. *Cancer*, **60**, 2682.

SCHOFIELD, P.N., CONNOR, H., TURNER, R.C. & ZAPF, J. (1989). Tumour hypoglycaemia: raised tumour IGFII mRNA associated with reduced plasma somatomedins. *Br. J. Cancer*, **60**, 661.

SHAPIRO, E.T., BELL, G.I., POLONSKY, K.S., RUBENSTEIN, A.H., KEW, M.C. & TAGER, H.S. (1990). Tumor hypoglycaemia: relationship to high molecular weight insulin-like growth factor II. *J. Clin. Invest.*, **85**, 1672.

TRICOLI, J.V., RALL, L.B., KARAKOUSIS, C.P. & 4 others (1986). Enhanced levels of insulin like growth factor messenger RNA in human colon carcinoma and liposarcomas. *Cancer Res.*, **46**, 6169.

VUK-PAVLOVIC, S., OPARA, E.C., LEVANAT, S., VRBANEC, D. & PAVELIC, K. (1986). Autocrine tumor growth regulation and tumor-associated hypoglycaemia in murine melanoma B16 *in vivo*. *Cancer Res.*, **46**, 2208.

WEBER, G., BANERJEE, G. & MORRIS, H.P. (1961). Comparative biochemistry of hepatomas. 1. Carbohydrate enzymes in Morris hepatoma 5123. *Cancer Res.*, **21,** 933.

WEBER, G. (1977). Enzymology of cancer cells. *New Eng. J. Med.*, **296,** 486.

YAMAMOTO, T., SEINO, Y., FUKIMOTO, H. & 7 others (1990). Over-expression of facilitative glucose transporter genes in human cancer. *Biochem. Biophys. Res. Commun.*, **170,** 223.